# Cost-effectiveness analysis of doctor-pharmacist collaborative prescribing for venous thromboembolism in high risk surgical patients

**DOI:** 10.1186/s12913-018-3557-0

**Published:** 2018-10-01

**Authors:** Andrew Hale, Greg Merlo, Lisa Nissen, Ian Coombes, Nicholas Graves

**Affiliations:** 1 School of Clinical Sciences, Faculty of Health, Queensland University of Technology, Royal Brisbane and Women’s Hospital, Cnr Butterfield St and Bowen Bridge Road, Herston, Brisbane, 4029 Australia; 2 Australian Centre for Health Services Innovation, School of Public Health and Social Work, Institute of Health and Biomedical Innovation, Queensland University of Technology, Kelvin Grove, Brisbane, 4059 Australia; 3 School of Clinical Sciences, Queensland University of Technology, Level 9, Q Block Room, 911, Brisbane, 4000 Australia; 4 Royal Brisbane and Women’s Hospital, Cnr Butterfield St and Bowen Bridge Road, Herston, Brisbane, 4029 Australia

**Keywords:** Pharmacist, Prescribing, Venous thromboembolism prophylaxis, Cost effectiveness, Pre admission clinic

## Abstract

**Background:**

Current evidence to support cost effectiveness of doctor- pharmacist collaborative prescribing is limited. Our aim was to evaluate inpatient prescribing of venous thromboembolism (VTE) prophylaxis by a pharmacist in an elective surgery pre-admission clinic against usual care, to measure any benefits in cost to the healthcare system and quality adjusted life years (QALYs) of patients.

**Method:**

A decision tree model was developed to assess cost effectiveness of pharmacist prescribing compared with usual care for VTE prophylaxis in high risk surgical patients. Data from the literature was used to inform decision-tree probabilities, utility, and cost outcomes. In the intervention arm, a pharmacist prescribed patient’s regular medications, documented a VTE risk assessment and prescribed VTE prophylaxis. In the usual care arm, resident medical officers were responsible for prescribing regular medications, and for risk assessment and prescribing of VTE prophylaxis. The base scenario assessed the cost effectiveness of a pre-existing pre-admission clinic pharmacy service that takes on a collaborative prescribing role. The alternative scenario assessed the benefits of introducing a pre-admission clinic pharmacy service where previously there had not been one. Probabilistic sensitivity analysis was conducted to explore uncertainty in the model.

**Results:**

In both the base-case scenario and the alternative scenario pharmacist prescribing resulted in an increase in the proportion of patients adequately treated and a decrease in the incidence of VTE resulting in cost savings and improvement in quality of life. The cost savings were $31 (95% CI: -$97, $160) per patient in the base scenario and $12 (95% CI: -$131, $155) per patient in the alternative scenario. In both scenarios the pharmacist-doctor prescribing resulted in an increase in QALYs of 0.02 (95% CI: -0.01, 0.005) per patient. The probability of being cost effective at a willingness to pay off $40,000 was 95% in the base scenario and 94% in the alternative scenario.

**Conclusion:**

Delegation of the prescribing of VTE prophylaxis for high risk surgical patients to a pharmacist prescriber in PAC, as part of a designated scope of practice, would result in fewer cases of VTE and associated lower costs to the healthcare system and increased QALYs gained by patients.

**Trial registration:**

Pre admission clinic study registered with ANZCTR-ACTR Number ACTRN12609000426280.

## Background

VTE causes a significant amount of death and disability worldwide, and is an extremely costly outcome for healthcare systems [1]. Venous thromboembolism (VTE) manifests as either deep vein thrombosis (DVT) or pulmonary embolism (PE), of which there were almost 15,000 cases in Australia in 2008, at a cost to the health system of $148 million [2]. Episodes of DVT or PE are costly to any healthcare system, estimated per case at $3000–9500 in the United States (US), and € 2000–4000 in Europe [3]. Development of VTE has been linked to several risk factors, including hospitalisation for acute medical illness or surgery [4]. Evidence has shown that appropriate use of prophylaxis using anticoagulants and/or mechanical devices reduces the development of VTE, and despite the production of evidence-based guidelines that outline this, these are often underutilised [5].

Pharmacist collaborative prescribing has been implemented as a model of care in to several healthcare systems internationally, including the United Kingdom (UK), and has replaced models where clinicians alone are responsible for prescribing [6]. It has the potential to improve Australian health services through improved access for consumers to effective medication usage, and alleviate current health workforce shortages by expanding the scope of the current health workforce through better utilisation of their skills [7–10]. It was suggested that future studies need to evaluate patient and health service outcomes, including economic analysis.

A single-centred randomised controlled trial in an elective surgery pre-admission clinic assessed the effectiveness of prescribing from a doctor-pharmacist collaborative prescribing model, including VTE prophylaxis, following a documented risk assessment [11]. The results showed that doctor-pharmacist collaborative prescribing produced medication charts that were as safe and accurate as usual care, and VTE prophylaxis was also as appropriate [11]. There were 95 patients at a high risk of VTE in the intervention arm, all of whom had their risk assessed and documented by the prescribing pharmacist. All 95 patients (100%) were prescribed appropriate VTE prophylaxis. In the usual care arm, there were 79 patients at a high risk of VTE, for whom the medical officers were responsible for assessing and prescribing VTE prophylaxis, of which 72 patients (91%) had appropriate VTE prophylaxis prescribed. Seven patients had anticoagulant prophylaxis inappropriately omitted from their medication chart, exposing them to a higher risk of DVT and PE. The potential impact of this improvement in prescribing by the pharmacist on health services efficiency is not known.

The aim of this analysis was to assess the cost effectiveness of having a doctor-pharmacist collaborative prescribing model compared with usual care in the prevention of VTE in high risk surgical patients, within the context of the Australian healthcare system.. This is the first study to assess the consequences of reallocating funding to support a doctor-pharmacist collaborative prescribing model for VTE prophylaxis in direct costs to the healthcare system and impact on patient quality of life.

The results of this analysis should enable a debate about changes in legislation in the Australian setting to allow pharmacist prescribing in collaborative models of care, that can improve cost effectiveness and quality of life for patients.

## Methods

**Fig. 1:**
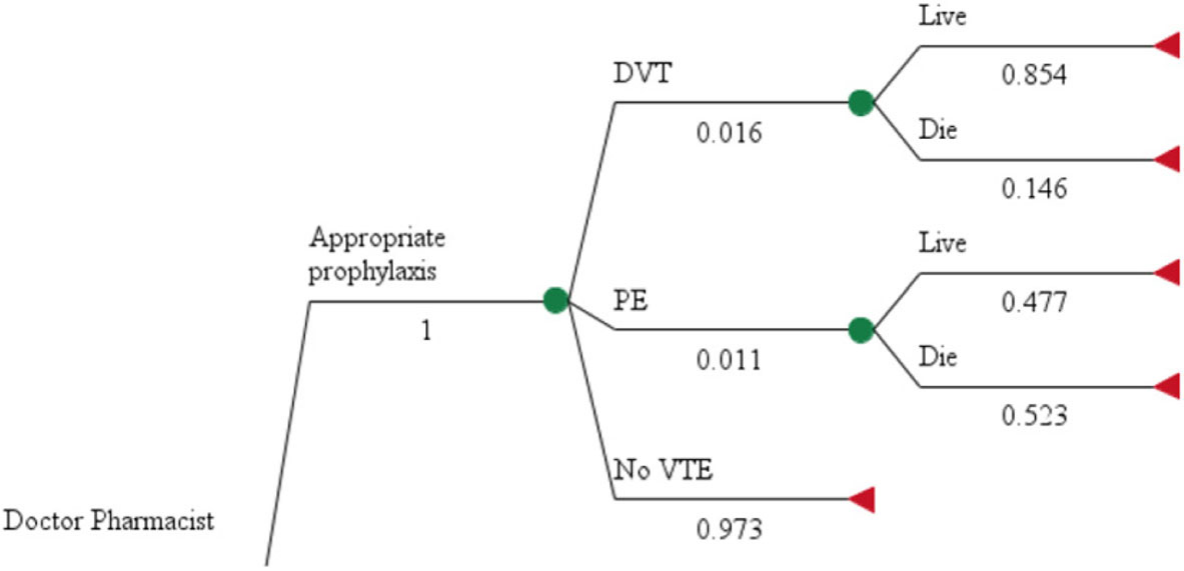
VTE Model Structure. See Table 1 for source of model parameter

A decision tree model was developed in TreeAge Pro [12] to assess the cost effectiveness of pharmacist prescribing compared with usual care for VTE prophylaxis in patients admitted to a pre-admission clinic for surgery (Fig. 1). Data from the literature were used to inform the decision-tree probabilities, utility, and cost outcomes (see Table 1). No patient subgroups were analysed.

**Table 1:** Summary of Model Parameters (including uncertainty at 95% confidence intervals)

Parameter	Value	Source
Mean number of patients in PAC per year (from 2009 to 2013)	5688	[11]
Number of patients at high risk of VTE	3583	[11]
Annual cost of new model of care Model 1 (existing pharmacy service)	$20,989	[11, 17]
Annual cost of new model of care Model 2 (new pharmacy service)	$154,579 - $12,741 = $141,838	[11, 17]
Appropriateness of VTE prophylaxis new model of care (%)	100 (SE 0.01)	[11]
Appropriateness of VTE prophylaxis usual care (%)	91.1 (84.7–97.3)	[11]
Probability of DVT with appropriate prophylaxis (%)	1.6 (0.9–2.3)	[11]
Probability of DVT with inappropriate prophylaxis (%)	4.0 (2.7–5.3)	[11]
Probability of PE with appropriate prophylaxis (%)	1.1 (0.7–1.5)	[11]
Probability of PE with inappropriate prophylaxis (%)	2.7 (1.9–3.3)	[11]
Probability of 12 months mortality post DVT (%)	14.6 (14.0–15.2)	[11]
Probability of 12 month mortality post PE (%)	52.3 (51.5–53.1)	[11]
Probability of 30 day mortality post DVT (%)	5.5 (5.1–5.9)	[11]
Probability of 30 day mortality post PE (%)	44.4 (43.6–45.2)	[11]
Direct cost to healthcare system of one episode of acute DVT	$10,077	[2]
Direct cost to healthcare system of one episode of acute PE	$10,042	[2]
QALYs lost post-acute episode of DVT	0.02 (0.01–0.04)	[16]
QALYs lost post-acute episode of PE	0.02 (0.01–0.05)	[16]
QALYs lost post-acute episode of DVT + 30 day death	0.0675 ()	[16]
QALYs lost post-acute episode of PE + 30 day death	0.0625 ()	[16]

The definitions of the interventions were based on those used in Hale et al. [11] In the doctor-pharmacist collaborative prescriber arm of the evaluation pre-admission clinic patients have their medications prescribed on the National Inpatient Medication Chart (NIMC) by a clinical pharmacist, and countersigned by a resident medical officer (RMO). The pharmacist also documents a VTE risk assessment. This contrasts with the usual care arm where medications are prescribed by the RMO alone. The scope of prescribing for the pharmacists is to continue or withhold regular medications, according to the plan for medications perioperatively, and to prescribe VTE prophylaxis according to local and national guidelines, following a risk and contraindication assessment.

For the main study, all patients who attended PAC and could provide written, informed consent were considered for participation. Patients were excluded if they were under 18 years of age, unable to communicate due to language difficulties or undergoing day surgery.

Patients were approached on arrival at clinic and written consent was obtained. After consent, patients were randomised using a computer generated randomisation list, in blocks of 10 (Microsoft Excel). Sealed envelopes (not prepared by the recruiting researcher) contained a zero or one as per the computer list; the next envelope was opened after consent to determine whether a patient entered the control or intervention arm respectively.

Patients in the two arms of the model have different probabilities of receiving appropriate prophylaxis according to guidelines. The effectiveness of pharmacist prescribing compared with usual care was based on the findings from Hale et al. [11] The findings from Hale et al. also defined the impact of having appropriate prophylaxis on reducing the risk of PE or DVT as defined in the model. Patients in the model who have PE or DVT have a risk of dying or living with reduced quality of life.

The number of surgical patient per year was calculated by taking the mean figure from annual numbers of patients in pre-admission clinic from the beginning of 2009, to the end of 2013. The incidence of high risk of VTE within this surgical population came from Hale et al. [11].

Cost of disease was estimated to be $10,077 per patient for DVT and $10,042 per patient for PE based on an Access Economics burden of disease study [2].

The benefit to patients of appropriate prescribing was measured in terms of quality adjusted life years (QALYs), due to the long term impact that appropriate prescribing potentially has on mortality and morbidity. Hogg et al. calculated utilities for DVT and PE via the standard gamble technique in patients with personal experience [13]. Median utility for acute DVT was 0.81 (interquartile range [IQR] 0.55–0.94) and acute PE 0.75 (IQR 0.45–0.91) QALYs were calculated by combining utility with estimated duration of symptoms. For both acute PE and acute DVT QALYs was the loss of QALYs associated with either PE or DVT was estimated to be 0.02 over the 2 year timeframe.

Cost-effectiveness was measured in terms of cost ($AU) per QALY. The evaluation takes an Australian healthcare sector perspective and does not consider costs outside this perspective. The time horizon of the study for the purpose of calculating QALY losses was the expected life of the patients. No discount rate was applied because the costs were assumed to be upfront.

### Cost scenarios

The analysis was undertaken using two different cost scenarios. The base scenario assesses the cost effectiveness of a pre-existing pre-admission clinic pharmacy service that takes on a collaborative prescribing role. The alternative cost scenario assesses the benefits in the introduction of a new pre-admission clinic pharmacy prescribing service where previously there had not been any pharmacist.

Resources required to deliver the doctor-pharmacist collaborative prescriber intervention include the time taken for the pharmacist to complete the medication chart offset by the time saved for the RMO not having to complete the medication chart. All other costs, for example nursing time, anaesthetist time and pathology testing remain constant and are not affected by the new model of care.

In the alternative cost scenario there is no pre-existing pharmacy service, so pharmacist collaborative prescribing pharmacist must be introduced. The cost of doctor-pharmacist collaborative prescribing in this scenario is estimated to be the annual salary plus on-cost of an HP level 5 pharmacist ($154,579pa), minus the cost of the time difference from the RMO no longer having to complete the medication chart as calculated in Model 1, per patient ($2.24 multiplied by the number of patients per year). The model assumes no extra cost due to space or equipment.

The only cost difference between usual care and the new model of care, is the increased pharmacist time taken to prescribe the NIMC, which would be offset to a certain degree by the reduction in RMO time required, due to no longer having to prescribe the NIMC. For each patient during the study, RMOs and pharmacists were requested to report the length of time taken for the patient to be seen, and mean appointment times were calculated from the patients with data entered **(see** Table 2).

**Table 2:** Appointment Times

(N)	Time (mins)	Time Difference (mins)
RMO Usual Care Arm (116)	26.78	
RMO Intervention Arm (123)	23.08	−3.6
Pharmacist Usual Care Arm (155)	17.67	
Pharmacist Intervention Arm (150)	24.26	+ 6.59

The hourly rates for the pharmacist and the RMO are based on Queensland health sources. The pharmacist is assumed to be HP level 5, to acknowledge the required postgraduate study and competencies required to develop and maintain the advanced scope of practice [14].

### Analysis

The differences in the cost and effectiveness of doctor-pharmacist collaborative prescribing compared with usual care were calculated and used to determine the incremental cost effectiveness ratio (ICER). The 95% confidence interval for the ICER was calculated through probabilistic sensitivity analysis using the Monte Carlo method with 1000 simulations. The findings of the sensitivity analysis were plotted onto a cost-effectiveness acceptability curve.

## Results

Based on the findings of Hale et al. it is estimated that the proportion of VTE patients who are high risk is 63%. The proportion of patients adequately treated was estimated to be 100% in the pharmacist prescriber arm and 91% in the usual care arm.

**Fig. 2:**
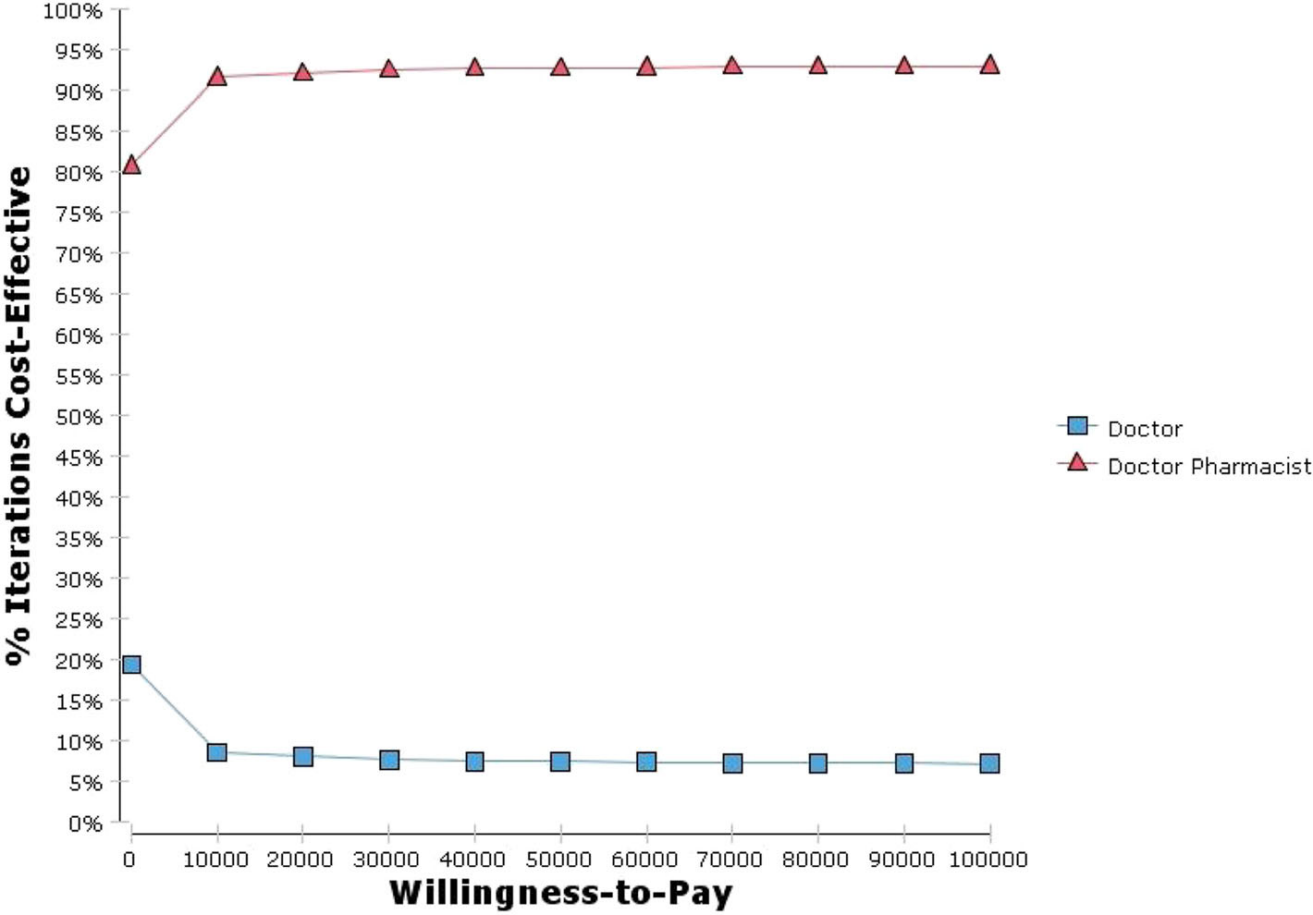
Cost effectiveness acceptability curve

In the base-case scenario pharmacist prescribing was non-significantly less costly than doctor prescribing by $31 (95% CI: -$97, $160) per patient compared with usual care and produced 0.02 (95% CI: -0.01, 0.05) QALYs per patient. Figure 2 presents the probability that pharmacist prescribing is cost effective as the willingness to pay increases. At a willingness-to-pay of $40,000 per QALY there is a probability of 95% that pharmacist prescribing is cost effective compared with usual care.

In the alternative scenario the cost saving for pharmacist prescribing was less than in the base scenario, with pharmacist prescribing saving $12 (95% CI: -$131, $155) per patient compared with usual care. There was a 94% probability that pharmacist prescribing is cost effective compared with usual care in the alternative scenario giving the willingness to pay of $40,000 per QALY.

## Discussion

Previous research has shown pharmacy interventions to be cost effective in contexts other than doctor-pharmacist collaborative prescribing for VTE prophylaxis. In the outpatient setting, Steele et al. showed face to face meetings between pharmacists and doctors, to discuss prescribing, significantly lowered prescription costs and achieved a higher saving than the pharmacist salary [15]. Patient outcomes were not assessed as part of the study. Elliott et al. assessed the cost effectiveness of a pharmacist telephone advisory line, aimed at improving adherence in patients with newly prescribed medications. The study concluded that the telephone service reduced non adherence in elderly medicine users, and reduced associated costs by reducing contact with healthcare providers [13, 16]. Karnon et al. undertook a cost-effectiveness analysis of various initiatives on admission to hospital, with a view to reducing the impact of potential medication errors. Pharmacy reconciliation on admission to hospital was the most cost-effective initiative, with highest impacts on the potential associated costs and patient harm [17]. In a review of non-medical prescribing from UK, the authors concluded that there were significant gaps in the literature to help evidence based policy making, and future research needs to include economic dimensions [9].

Findings from this decision analytic model demonstrated an improvement in the appropriateness of prescribing of VTE prophylaxis in the pharmacist prescriber arm, in patients who needed it. And in turn a decreased incidence of adverse events as well as a decrease in the cost and quality of life impacts associated with these adverse events. An increased appropriateness in prescribing resulted in a decreased probability of the patient suffering an event in the peri-operative period, and a decreased chance of the ongoing costs associated, both in terms of financial costs to the health service and health costs to the patient.

It is important to acknowledge that this is a collaborative model of care, and whilst the pharmacist makes the initial risk assessment, and decision on prescribing of prophylaxis, the decision needs to be confirmed by the RMO in clinic. Likewise, it is important for the prescribing pharmacist to recognise limitations, and refer any patients that may fall outside of guidelines to the medical officer for further consideration of appropriate prescribing.

It is also important to acknowledge the need to continue and focus on junior doctor education, with a view to improving the appropriateness of prescribing within the usual care arm, as it has been suggested that the same results could be obtained by providing the junior doctors with extra prescribing training. The evidence for the effectiveness of pharmacist prescribing comes from a single study—which was the only study to assess the effectiveness of pharmacist prescribing compared with usual care for VTE prophylaxis in presurgical patients. The trial, and also this economic model uses a specific model of care for pharmacist prescribing. The future challenge lies in ensuring that any training courses and practical training is appropriate, so that the improvements in the appropriateness of the prescribing demonstrated in this scope of practice can be replicated on a larger scale, and across other scopes.

The economic model assumes probabilities of a VTE event post prescribing, taken from the literature. Any study that looks at actual DVT or PE as clinical outcomes as an endpoint, requires data outside of the scope of our study.

## Conclusion

Delegation of the prescribing of VTE prophylaxis for high risk surgical patients to a pharmacist prescriber in PAC, under a collaborative prescribing model and as part of a designated scope of practice, would result in fewer cases of VTE and associated lower costs to the healthcare system and increased QALYs gained by patients.
